# The state of rice value chain upgrading in West Africa

**DOI:** 10.1016/j.gfs.2020.100365

**Published:** 2020-06

**Authors:** Guillaume Soullier, Matty Demont, Aminou Arouna, Frédéric Lançon, Patricio Mendez del Villar

**Affiliations:** a CIRAD, UMR ART-DEV, F-34398, Montpellier, France; b ART-DEV, Univ Montpellier, CIRAD, CNRS, Univ Montpellier 3, Univ Perpignan Via Domitia, Montpellier, France; c International Rice Research Institute (IRRI), Los Baños, Laguna, Philippines; d Africa Rice Center (AfricaRice), 01 BP 2551, Bouake 01, Cote d’Ivoire; e CIRAD, UMR TETIS, F-34398, Montpellier, France; f TETIS, Univ Montpellier, AgroParisTech, CIRAD, CNRS, IRSTEA, Montpellier, France

**Keywords:** Rice, Value chain, Upgrading, Africa, Contract farming, Milling

## Abstract

Following the food price crisis in 2008, African governments implemented policies aiming at crowding in investment in rice value chain upgrading to help domestic rice compete with imports. We assess the state of rice value chain upgrading in West Africa by reviewing evidence on rice millers’ investment in semi-industrial and industrial milling technologies, contract farming and vertical integration during the post-crisis period 2009–2019. We find that upgrading is more dynamic in countries with high rice production and import bills and limited comparative advantage in demand. However, scaling of upgrading faces several challenges in terms of vertical coordination, technology, finance and policies. Our assessment can help value chain actors and policy makers refine upgrading strategies and policies to increase food security in West Africa.

## Highlights

• Rice value chain upgrading in West Africa is heterogeneous among countries.
• Upgrading involves milling technologies, contract farming and vertical integration.
• In 2019, 57 industrial and semi-industrial rice mills were operating in West Africa.
• Import bill, rice production and cultural heritage jointly explain 89% of heterogeneity.
• Challenges include vertical coordination, technology, finance and policies.

## 1 Introduction

The food price crisis in 2008 redirected international attention towards domestic food value chains’ (VCs) capacity and resilience in providing food security in developing countries (World Bank, 2008). In West Africa, the attention turned towards rice VCs because rice is the most important calorie source in this region (Macauley and Ramadjita, 2015). To address chronic hunger through macro-nutrient self-sufficiency, African policy makers developed targeted National Rice Development Strategies (NRDS) under the Coalition for African Rice Development (CARD, 2019). However, while domestic rice production increased after the crisis, domestic rice VCs never managed to catch up with consumption, leading to an increasing gap that is satisfied through imports (Mendez del Villar and Lançon, 2015). Therefore, policy makers were urged to revisit their *productivist* NRDS and create a favorable enabling environment for crowding in private sector investment in VC upgrading (Demont, 2013).

A decade after the 2008 food price crisis, it is time to make an assessment of the current state of rice VC upgrading in West Africa. Are domestic rice VCs being upgraded in this region, and if they are, what type of investments have been conducted and where? In particular, there is little information about investments in new processing technologies that would help domestic rice compete with imports quality- and cost-wise. Therefore, this paper attempts to document the technological and coordination changes that have been implemented at processing level in rice VCs in West Africa over the last decade. In particular, we compile and review evidence of public and private investment in upgraded processing facilities, contract farming schemes and vertical integration in 15 West African countries. We also assess the opportunities and challenges encountered in rice VC upgrading. Our assessment may help policy makers at national and regional levels and VC actors revisit and refine upgrading strategies and policies during the revision of the NRDS under the CARD Phase 2, which aims at doubling rice production in Sub-Saharan Africa from 28 million tons in 2019 to 56 million tons by 2030 (CARD, 2019).

## 2 Method

To identify, collect and validate evidence of investment in rice VC upgrading in the 15 West African countries (Table 2), we followed three stages. First, we conducted a non-systematic review of peer-reviewed and non-peer reviewed literature. We initiated our literature review with a focused search of economic studies through Econlit using the following keywords: rice; value chain; investment; mill; processing; contract; vertical integration; and the names of the 15 West African countries. The keywords aimed at identifying investments in semi-industrial and industrial milling technologies and in contract farming and vertical integration, implemented during the decade of 2009–2019 in the wake of the food price crisis. We then expanded our search to cover multiple disciplines by using Web of Science® and Google Scholar®. To identify non-peer reviewed evidence such as reports from development organizations, we further broadened our search by using Google® and Bing®. We drew significant insights from the study on rice VCs in Africa that was carried out by the Food Fortification Initiative and the Global Alliance for Improved Nutrition (FFI and GAIN, 2017). Secondly, to complete our literature review and validate the evidence, we drew on personal expertise and consultation of experts through the authors' professional network of partners established under the CGIAR Flagship Project on “Upgrading Rice Value Chains” (http://ricecrp.org). Thirdly, we presented our final assessment at (i) the Fifth International Rice Congress, Singapore, 14–17 October 2018; and (ii) the regional workshop on “Leveraging small and medium rice millers for rural transformation and investment in the rice sector in Africa,” jointly organized by the Food and Agriculture Organization of the United Nations (FAO), the Africa Rice Center and the Rice Council of Tanzania in Dar es Salaam, Tanzania, 28–30 May 2019 (http://ricecrp.org/news). At the workshop, the evidence was validated by 48 participants representing public and private sectors from nine African countries (Benin, Cameroon, Côte d’Ivoire, Kenya, Mali, Nigeria, Senegal, Tanzania, and Uganda) (Arouna, 2019). To the best of our knowledge, the resulting evidence compiled in Table 2 can be considered to be quasi-exhaustive and representative of the current state of rice VC upgrading in West Africa.

To analyse the evidence, we first measured *dynamism* of rice VC upgrading in the 15 West African countries through four outcome indicators: (i) number of investments in semi-industrial and industrial mills that were operational in 2019; (ii) aggregate upgraded milling capacity; (iii) the number of farmers involved in contract farming; and (iv) the area under vertical integration. We further traced the origin of investment as an intermediate outcome indicator capturing the extent to which rice sectors crowd in public investment (PI), foreign direct investment (FDI) or domestic private investment (DPI). Secondly, we compiled a list of factors that could favour and hinder investment in modern milling technologies and ran a linear regression analysis to identify the main factors that explain heterogeneity of rice VC upgrading among the 15 West African countries.

## 3 Background: upgrading domestic rice value chains to improve food security in West Africa

### 3.1 Rice for food security in West Africa

Food insecurity remains an issue in West Africa. At the global level, the number of undernourished people (i.e. people whose dietary energy consumption is below their dietary energy requirement) decreased from 841.7 million in 2009 to 821.6 million in 2018 (FAO, 2019). However, the number of undernourished people in West Africa increased from 31.5 million to 56.1 million over the same period, representing 14.7% of the West African population in 2018. Between 2009 and 2018, there were on average 15.7 million of undernourished people in Nigeria, and at least one million people undernourished in almost each other West African country (Table 1).

**Table 1:** Main indicators for food security and rice sector in West Africa.

Country/Region	Food Security					Rice sector									
						Production				Imports		Consumption		Import dependency	
	Average annual number of people undernourished, 2009–2018 (million)	Average annual growth of the number of undernourished people, 2009–2018 (%)	Average annual rice consumption, 2009–2013 (kg/capita/year)	Average annual growth of rice consumption, 2009–2013 (%)	Average daily energy consumption through rice, 2009–2013 (kilocalories/capita/day)	Average annual production (milled equivalent), 2009–2019 (10^3^ ton)	Average annual growth of production, 2009–2019 (%)	Average annual area, 2009–2019 (10^3^ ha)	Average annual number of rice growers, 2009–2019	Average annual imports, 2009–2019 (10^3^ ton)	Average annual growth of imports, 2009–2019 (%)	Average annual consumption, 2009–2019 (10^3^ ton)	Average annual growth of consumption, 2009–2019 (%)	Average annual import dependency, 2009–2019	Average annual growth of import dependency, 2009–2019 (%)
World	821.6	−0.4	54	0.0	543	478,319	1.2	160,996		39,243	4.3	467,996	1.2	0.08	4.32
Sub-Saharan Africa	199.0	3.0	23	1.4	232	15,402	6.8	11,514	8,053,309	12,409	7.8	27,249	6.8	0.46	1.26
West Africa	39.1	7.1	39	1.0	381	10,098	10.1	7565	4,131,103	8205	7.0	17,986	8.1	0.46	−1.09
Benin	1.1	0.0	55	−0.7	208	142	13.5	67	78,063	411	37.1	542	22.2	0.76	1.22
Burkina	3.5	1.7	21	−0.7	208	204	6.2	144	177,653	395	15.4	590	12.4	0.67	1.40
Cote d'Ivoire	4.6	0.8	64	−0.3	578	1111	19.4	731	838,962	1184	7.1	2180	9.4	0.54	4.30
Gambia, The	0.2	0.0	66	−1.8	649	35	−5.9	67	32,056	168	15.8	200	12.9	0.84	1.84
Ghana	1.6	2.6	32	1.8	301	359	9.1	220	43,985	655	13.6	986	10.6	0.66	2.48
Guinea	1.9	1.2	97	−0.4	983	1300	5.5	1342	965,658	566	16.5	1752	8.3	0.32	1.08
Guinea-Bissau	0.4	7.4	95	1.5	948	108	−0.3	104	87,870	135	4.1	241	1.9	0.56	−3.89
Liberia	1.6	3.2	91	1.2	914	172	−1.3	233	182,088	293	7.4	455	3.9	0.64	0.13
Mali	1.0	1.0	57	−0.4	567	1442	16.3	730	405,228	188	15.5	1627	12.9	0.12	−4.01
Mauritania	0.4	7.4	43	4.0	423	129	42.0	42	10,934	111	2.7	234	11.6	0.47	−1.05
Niger	2.3	9.9	12	0.4	111	61	3.1	19	7121	295	4.2	357	4.0	0.83	19.23
Nigeria	15.7	18.8	28	2.3	284	3736	10.8	2967	487,573	2359	−0.9	6032	5.5	0.39	5.94
Senegal	1.8	0.7	71	0.9	698	508	18.8	198	186,631	981	6.8	1527	8.9	0.64	−0.59
Sierra Leone	1.7	0.6	99	2.0	929	702	3.9	625	559,304	295	45.5	1006	8.4	0.29	0.57
Togo	1.3	−0.8	23	3.7	233	90	1.5	75	41,063	168	25.0	256	11.5	0.66	2.75

*Notes*: Import dependency is calculated as the share of consumption covered by imports.

*Sources*: Data on food security retrieved from FAO (2019); data about the rice sector retrieved from USDA (2019), except for the number of rice growers that was calculated by applying the rural populations' growth rates estimated by FAO and AfDB (2015) to the 2009 estimate of Diagne et al. (2013).

In West Africa, rice is a strategic commodity for food security. Rice consumption quickly expanded since the 1960s driven by demographic growth, rising per capita consumption and urbanization (Mendez del Villar and Lançon, 2015). The annual per capita rice consumption steadily increased from 10 kg in 1961 to 54 kg in 2017 (USDA, 2018). Between 2009 and 2013, rice consumption was more important in West Africa than in any other region of the continent. In particular, the highest rice consumption rates were observed in Guinea, Guinea-Bissau, Liberia and Sierra Leone (more than 90 kg per capita per year), and in Senegal, Benin, Côte d’Ivoire, The Gambia and Mali (more than 50 kg per capita per year) (Table 1). Furthermore, rice consumption rapidly increased in Nigeria (2.3% per year) and Ghana (1.8%), the two most populated countries in the region. Rice therefore becomes an increasingly important source of calories in West Africa. The average energy contributed by rice increased from 367 to 384 kcal per capita and per day between 2009 and 2013. This makes rice a strategic commodity to tackle food insecurity in the region.

Since the independence, rice production is rapidly increasing in West Africa. The production steadily increased from around 2.2 million tons in 1962 to 12.7 million tons in 2018 (USDA, 2019). Over the 2009–2019 decade, West Africa produced an average of 10.1 million tons of rice annually, representing 65.6% of the total Sub-Saharan African production (Table 1). The main producers in West Africa were Nigeria (3.7 million tons), Mali (1.4 million tons), Guinea (1.3 million tons) and Côte d’Ivoire (1.1 million tons). Furthermore, rice production continues to grow. Over the last decade, the average annual growth rate of rice production was 10.1%. The countries that mostly contributed to this increase were Nigeria, Senegal, Mali, Ghana and Côte d’Ivoire, where production increased between 9.1% and 19.4% per year. This increase was fuelled by an increase in rice areas (7.5% per year). On the contrary, yield gains did not contribute much to production increase, because of low adoption of improved varieties and lack of good quality seed (Arouna et al., 2017a), low use of inputs and low adoption of good agricultural practices.

However, West Africa faces a structural rice deficit, and the region increasingly relies on imports. Indeed, the increase in production is not able to keep pace with the sharp increase in rice consumption (USDA, 2019). The share of imported rice in total consumption increased from 20% in the 1960s to 46% in 2009. This ratio slightly decreased between 2009 and 2019, due to small reductions in imports recorded in Senegal, Mali, Mauritania and Guinea-Bissau. However, rice import dependency increased in all other West African countries over the same period. The main importers were Nigeria (2.4 million tons), Côte d’Ivoire (1.2 million tons) and Senegal (1 million tons). West Africa therefore remains the second biggest rice importer in the world, after China.

### 3.2 Policies to upgrade domestic rice value chains in West Africa

Domestic rice in West Africa is mainly supplied by traditional VCs. These VCs proliferated after liberalization and the decrease in state control of industrial mills. They are often made of several intermediaries owning little capital and managing small quantities (Reardon et al., 2014). Traditional millers tend to purchase paddy through spot transactions, which do not include quality criteria and incentives for proper moisture rates, impurity rates, and varietal homogeneity. The traditional technologies used by millers include manual milling, as well as the simple huller, *monobloc* (one-pass mill) and *minirizeries.* As a result, traditional millers generally produce rice of low quality and purity featuring a heterogeneous mix of varieties and high rates of broken grains, leading to low cooking quality of the product. These traditional VCs have difficulties competing against structured import VCs in terms of quality, cost and scale (Soullier and Moustier, 2013).

**Fig. 1:**
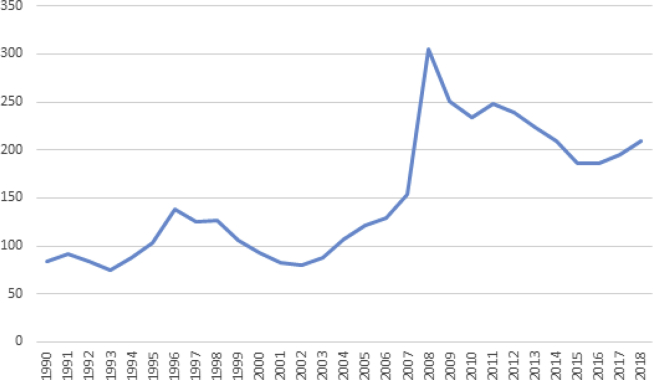
Average global rice price (US$/t f.o.b.). *Note:* Prices are free on board (f.o.b.), weighted by their share of the total rice internationally traded. *Source:* OSIRIZ/InfoArroz (2018).

The food price crisis in 2008 revealed that global market dependence was a risk for national food security. Within a few weeks, the rice price increased threefold, and remained steady at a higher level than prior to the crisis (Fig. 1). This led to protests in several major cities, and some of the poorest and most vulnerable households became food insecure (Cudjoe et al., 2010; Kumar and Quisumbing, 2013). This food price crisis was a major incentive for policies to upgrade domestic rice VCs (Seck et al., 2010). In order to improve food security in West Africa, African policy makers developed targeted NRDS under the Coalition for African Rice Development (CARD, 2019). The purpose was to replace imports by domestic rice.

The literature on VCs defines upgrading as the process of acquiring new skills and accessing new markets through participation in a particular VC (Humphrey, 2004). In West Africa, policies mainly aim at upgrading rice VCs through technical and organizational changes, in order to increase the quantity produced and decrease production costs through economies of scale. Policies targeted an increase in paddy production by providing small-scale producers with access to improved inputs and increasing the area planted in rice through land developments (Demont, 2013). Policies also encouraged and supported investments in semi-industrial or industrial milling technologies. Indeed, these technologies perform functions that dramatically improve the quality of milled rice. These functions include pre-cleaning, drying, cleaning, stone picking, weighting, hulling, separating, whitening, grading and bagging. Two broad types of upgraded technologies exist. The semi-industrial milling technology is a milling line performing at least four quality upgrading functions and with a theoretical capacity ranging between two and three tons of paddy per hour. The industrial technology is a milling line performing at least six quality upgrading functions and with a theoretical capacity ranging between three and five tons of paddy per hour. Investments in such technologies often include large storage capacities. The investments in these upgraded technologies were promoted by international organizations (CARD, 2008; World Bank, 2008), and several states created national agencies supporting foreign investors (Tyrou et al., 2019). Some policies also supported traditional millers to upgrade their technologies through subsidies.

The companies managing upgraded technologies need to collect high volumes of paddy to achieve economies of scale, and to collect paddy of good quality. Indeed, the quality of paddy, defined in terms of cleanliness, moisture content, varietal purity and homogeneity, strongly influences milled rice quality. For these reasons, upgraded mills can deploy vertical coordination modes with rice growers. Millers can indirectly control paddy production through contract farming, defined as “a sales arrangement between a farmer and a firm, agreed before production begins, which provides the farmer with resources or services” (Ton et al., 2018). Contract farming may include the supply and/or prefinancing of improved inputs, credit and technical advice, and quality criteria rewarded through price premiums. Such access to improved input and quality markets can improve farmers’ income (Bellemare and Bloem, 2018; Bellemare and Lim, 2018; Ton et al., 2018; Arouna et al., 2017b). Alternatively, millers can directly control paddy production. In this arrangement, which is often called vertical integration or hierarchy, millers employ workers to grow rice on their fields and maintain direct administrative control over agricultural production.

### 3.3 Cultural and physical barriers to rice imports

Upgrading domestic rice VCs in West Africa is a challenge because consumers in coastal countries and cities generally prefer imported rice, with the exception of those close to secondary centers of origin of rice domestication such as Guinea, Sierra Leone, The Gambia and the Casamence region in Senegal (Demont, 2013; Demont et al., 2017; Demont and Ndour, 2015). However, research based on framed field experiments has revealed that domestic rice can compete with imported rice if its quality is tailored to urban consumer preferences (Demont et al., 2017; Demont and Ndour, 2015). Furthermore, in the competition against imports, two comparative advantages “shield” countries from world market pressures and mitigate exposure to rice imports to some extent. First, countries' remoteness from a seaport and landlocked status can act as a *physical* barrier and mitigate exposure to imports as it increases the competitiveness of locally produced rice relative to imported rice (Demont, 2013; Demont and Ndour, 2015). Secondly, countries’ proximity to the primary (middle Niger delta in Mali) and secondary centers of origin of rice domestication (Fouta Djallon highlands in Guinea and the Gambia and Casamance rivers) tend to preserve indigenous preferences for local rice. Hence, proximity to origin of rice domestication can raise *cultural (preference)* barriers to rice importation as consumers tend to be more attached to local rice due to an ingrained 3000-year-old tradition of producing and consuming rice locally. In other words, preference for local rice endows local rice sectors with a “comparative advantage in demand,” which reduces the competitive pressure to upgrade rice VCs (Demont, 2013; Demont et al., 2017; Demont and Ndour, 2015). The absence of a comparative advantage of demand, on the other hand, tends to make rice sectors more vulnerable to import pressure, which triggers competitive responses in terms of investments in varietal quality, processing technologies, improved packaging and labelling.

## 4 Evidence of rice value chain upgrading in West Africa

In Table 2, we present the status of rice VC upgrading in the 15 West African countries through the four outcome indicators presented in the method, the intermediate outcome indicator of the origin of investment, as well as the factors that were retained for the regression analysis aiming at explaining heterogeneity of upgrading (Table 3 and Table 4). Based on the outcome indicators, we classify the countries into three groups, differentiating between countries where the evidence suggests that rice VC upgrading is (i) dynamic, (ii) moderate or (iii) inexistent. The evidence compiled in Table 2 suggests that there were 57 operating rice mills with semi-industrial or industrial technologies in West Africa in 2019, amounting to an aggregate capacity of 315 tons per hour. We found evidence of contract farming in eight countries, involving a minimum of 10,890 producers, and vertical coordination in five countries, covering at least 29,240 ha.

**Table 2:** State of rice value chain upgrading in 15 countries in West Africa, 2009–2019.

Country	Number of investments that were operational in 2019	Aggregate upgraded milling capacity (tons per hour)	Origin of investments	Vertical coordination				Exposure to imports		Average annual milled rice production, 2009–2019 (10^3^ tons)	Source
				Contract farming (number of farmers)	Share of contracted farmers (%)	Vertical integration (hectares)	Share of area under vertical integration (%)	Import barriers^a^	2008 import bill (10^6^ US$)^b^		
*Group 1: Dynamic rice value chain upgrading*											
Nigeria	24 industrial mills	177	FDI, DPI	3000	0.61	20,400	0.69	None	772	3736	^c,d,e^
Senegal	15 industrial and semi-industrial mills	60	FDI, DPI	3500	1.85	3590	1.86	None	645	508	^c,f^
*Group 2: Moderate rice value chain upgrading*											
Ghana	1 industrial mill, 3 semi-industrial mills	26	FDI, DPI	4000	9.09	750	0.34	None	216	359	^c,g,h,i^
Mali	4 industrial mills	20	FDI, DPI	–	–	3200	0.44	Physical, cultural	66	1442	^j,k^
Côte d’Ivoire	2 industrial mills, 1 semi-industrial mill	15	PI, DPI	10 (experimental)	0.00	–	–	Cultural	472	1111	^l^
Burkina Faso	1 industrial mill, 1 semi-industrial mill	7	DPI	140	0.08	–	–	Physical	56	204	^m,n,o,p^
Liberia	2 semi-industrial mills	4	DPI, PI	–	–	–	–	None	75	172	^c,s^
Niger	2 semi-industrial mills	4	PI	–	–	–	–	Physical	126	61	^p,q,r^
Sierra Leone	1 semi-industrial mill	2	DPI	–	–	1300	0.21	Cultural	85	702	^c^
Benin	17 ESOP	–	DPI	140	0.18	–	–	None	185	142	^c,t,u,v^
Togo	15 ESOP	–	DPI	100	0.24	–	–	None	9.3	90	^c;t,v^
*Group 3: No rice value chain upgrading*											
Guinea	–	–	–	–	–	–	–	Cultural	153	1300	^c^
Mauritania	–	–	–	–	–	–	–	None	77	129	^c^
The Gambia	–	–	–	–	–	–	–	Cultural	28	35	^c^
Guinea-Bissau	–	–	–	–	–	–	–	Cultural	10	108	^c^
West Africa	57 upgraded units	315		10,890	0.26	29,240	0.39		2975.3	10,098	

*Notes*: The evidence of investments in semi-industrial and industrial technologies and implementation of vertical coordination needs to be interpreted as being relative to the baseline, which consists of traditional millers purchasing paddy from traders on spot markets or interlinked transactions. Only operating units and installed over the last decade are reported; unfinished investments or units that had terminated their operations were not considered as evidence of upgrading. Semi-industrial mills can theoretically process between two and three tons of paddy per hour and perform at least four quality upgrading functions. Industrial mills can theoretically process between three and five tons of paddy per hour and perform at least six quality upgrading functions. Traditional mills process under two tons of paddy per hour. *Entreprises de Services et Organizations de Producteurs* (ESOPs) are traditional processing units in which farmers have the opportunity to gradually become shareholders. FDI: foreign direct investment; DPI: domestic private investment; PI: public investment. Dashes indicate absence of evidence of operating upgraded technologies or vertical coordination.

Sources: ^a^Demont (2013), Demont and Ndour (2015) and Demont et al. (2017); ^b^IRRI (2019); ^c^FFI and GAIN (2017); ^d^Awotide et al. (2015); ^e^Hathie (2016); ^f^Soullier and Moustier (2019); ^g^Ayeduvor (2018); ^h^Bidzakin et al. (2018); ^i^Bannor et al. (2017); ^j^Coulibaly and Havard (2015); ^k^Coulibaly and Soullier (2020); ^l^(Soullier et al., 2019); ^m^Bila (2015); ^n^Tapsoba (2016); ^o^(Sirdey et al., 2018); ^p^VECO (2014); ^q^ (Fall 2016); ^r^RiceHub (2016); ^s^Oxfam (2018); ^t^ETD (2016); ^u^Maertens and Vande Velde (2017); ^v^Adabe et al. (2019).

**Table 3:** Determinants of aggregate upgraded milling capacity in 15 countries in West Africa (linear regression).

Variable	Coefficient	SE	P-value
2008 import bill (10^6^ USD)	0.058	0.030	0.077*
Average annual milled rice production (2009–2019, 10^3^ tons)	0.032	0.007	0.001***
Cultural import barriers	−24.660	9.613	0.028**
Physical import barriers	−2.769	11.251	0.811
Constant	−1.780	8.169	0.832

*Notes:* Sample size = 15; R^2^ = 0.911; Adjusted R^2^ = 0.875; SE: standard error. Cultural and physical import barriers are captured through dummies. Variance inflation factors (VIF) are in the range of 1.15–2.61 with a mean VIF of 1.84. A Breusch-Pagan/Cook-Weisberg test for heteroscedasticity generates a P-value of 0.788. Significance levels: *p < 0.1; **p < 0.05; ***p < 0.01.

*Source:* Data compiled in Table 2.

**Table 4:** Determinants of aggregate upgraded milling capacity in 15 countries in West Africa (stepwise linear regression).

Variable	Coefficient	SE	P-value
2008 import bill (10^6^ USD)	0.061	0.026	0.042**
Average annual milled rice production (2009–2019, 10^3^ tons)	0.032	0.006	0.000***
Cultural import barriers (dummy)	−24.168	8.992	0.021**
Constant	−2.773	6.792	0.691

*Notes:* Sample size = 15; R^2^ = 0.910; Adjusted R^2^ = 0.886; SE: standard error. Cultural and physical import barriers are captured through dummies. Variance inflation factors (VIF) are in the range of 1.20–2.29 with a mean VIF of 1.90. A Breusch-Pagan/Cook-Weisberg test for heteroscedasticity generates a P-value of 0.774. Significance levels: *p < 0.1; **p < 0.05; ***p < 0.01.

*Source:* Data compiled in Table 2.

The regression analysis identifies three factors that jointly explain 89% of heterogeneity in aggregate upgraded milling capacity in the 15 countries: (i) the 2008 import bill (total value of rice imports, expressed in million US$) (IRRI, 2019); (ii) the average annual domestic milled rice production in 2009–2019 (FAO, 2019); and (iii) cultural (preference) import barriers (Table 3, Table 4).^1^ The 2008 import bill (total value of rice imports by a country) is a measure for the pressure policy makers may have experienced to decrease reliance on the world market at the height of the rice price crisis, right before the period of our analysis (2009–2019). Whereas the import bill measures the market opportunities for investors from the demand side in terms of the demand gap that can be closed through domestic production, the annual production levels measure the market opportunities from the supply side in terms of paddy availability. Both drivers provided strong incentives for private sector investment.

Table 2 suggests that upgrading tends to be more dynamic when countries and rice sectors are able to attract FDI. It is worthwhile noting that only *cultural* (preference) import barriers were found to significantly slow down investment and not *physical* import barriers (Table 3, Table 4). This was predicted by Demont (2013), who argued that the extent to which landlockedness reduces exposure to imports strongly depends on cross-border trade infrastructure and relationships with neighboring coastal countries (Faye et al., 2004).

Group 1 includes countries where rice VC upgrading was found to be most dynamic among all countries, i.e. Nigeria and Senegal (Table 2). The domestic rice sectors in these coastal countries hosting big seaports (Lagos and Dakar) were exposed to the highest rice import bills in 2008 (above 500 million US$) without benefiting from any mitigating physical or cultural barriers. As a result, these countries were politically most pressured to upgrade their domestic rice VCs. Nigeria offered the greatest economic opportunities for rice millers in terms of market size and paddy availability. Indeed, it featured the largest demand (on average 6.0 million tons of rice consumption per year between 2009 and 2019) (USDA, 2019), and the largest paddy supply in West Africa (on average 3.7 million tons of milled rice production per year between 2009 and 2019). In Senegal, average annual rice consumption was 1.5 million tons over the same period (USDA, 2019) and milled rice production averaged 0.5 million tons (Table 1). The Senegalese government, supported by international organizations, implemented attractive policies for investment, notably through the creation of a national agency promoting foreign investment (Soullier et al., 2018).

In 2019, there were 24 industrial mills operating in Nigeria and 15 industrial and semi-industrial mills operating in Senegal (FFI and GAIN, 2017; Soullier and Moustier, 2019). Most of these mills were built since 2009 (Awotide et al., 2015). They were owned by foreign private companies, such as the groups Stallion and Olam in Nigeria, or ASI in Senegal. Other mills were owned by national actors, such as importers (FFI and GAIN, 2017) or small-scale millers that upgraded their technology (Soullier and Moustier, 2019). Some of these companies were established with financial support from international organizations, such as the CNT mill in Senegal (Soullier and Moustier, 2019). In 2014, Senegalese millers processed each between 7000 and 18,000 tons of paddy and were implementing branding strategies (Soullier and Moustier, 2019).

To sustainably source reliable volumes of paddy, millers in both countries in Group 1 adopted vertical coordination modes. In 2014, three rice millers in Senegal used production contracts to source 15,000 tons of paddy grown on 3500 ha by 1500 producers (Soullier and Moustier, 2019). The contracts included supply and prefinancing of seed, fertilizer, herbicides and sometimes technical advice. Farmers reimbursed the inputs through paddy, and the contracts included quality criteria (moisture content and impurity rates) (Soullier and Moustier, 2018). Furthermore, in 2014, five upgraded mills in Senegal purchased 15,000 tons of paddy from 2000 producers growing 4000 ha of rice through marketing contracts (Soullier and Moustier, 2019). These contracts specified quality criteria, but did not include input supply. The price was negotiated within the interprofessional association. In Nigeria, there were at least three industrials mills (Olam, Veetee and Ebony Rice) that also sourced their supplies through production contracts (Awotide et al., 2015). In the Nasarawa state, around 3000 rice growers were contracted by Olam (Hathie, 2016). In both countries, millers also integrated rice production. In this system, the land could be rented to public agencies, local councils or farmers, and the millers F controlled paddy production. In Nigeria, rice millers controlled areas ranging from 400 to 10,000 ha (FFI and GAIN, 2017). In Senegal, the areas ranged from 20 to 2600 ha (Soullier et al., 2018).

Group 2 includes West African countries that faced rice import bills below 500 million US$ in 2008 and where VC upgrading was slowly emerging between 2009 and 2019, i.e. Ghana, Mali, Côte d’Ivoire, Burkina Faso, Liberia, Niger, Sierra Leone, Benin and Togo. Some countries in this group are fully exposed to the world market (Ghana, Benin, Liberia and Togo). The others benefit from either physical barriers due to their landlocked status (Niger and Burkina Faso), cultural barriers due to *geographical* (Sierra Leone is close to Fouta Djallon in Guinea) or *genealogical*^2^ (Côte d’Ivoire) proximity to rice cultural heritage, or both (Mali) (Demont et al., 2017). Furthermore, most countries in this group feature lower production levels than countries in Group 1. Despite high production levels in Côte d’Ivoire (averaging 1.1 million tons of milled rice per year during 2009–2019) and Mali (1.4 million tons) (Table 1), political crisis and conflicts in these countries may have slowed down investment, in addition to reduced exposure to imports thanks to the cultural and physical barriers mentioned earlier.

Although some of these countries were able to crowd in FDI (Ghana and Mali), domestic private companies carried out most of the investments. In Ghana, the foreign private company Avnash built a mill with a capacity of 20 tons of paddy per hour to market parboiled and white rice (Ayeduvor, 2018; FFI and GAIN, 2017). Furthermore, three mills with capacities averaging two tons per hour invested and processed between 3000 and 9000 tons of paddy per year (FFI and GAIN, 2017). In Mali, four industrial mills were created since 2011 (Coulibaly and Soullier, 2020). They collect paddy in the Niger Office and use several milling lines with theoretical capacities ranging between 3 and 5 tons per hour (Coulibaly and Soullier, 2020). In Côte d’Ivoire, in 2016 an importer invested in semi-industrial milling and developed the brand *Le Fromager* (Soullier et al., 2019). In Burkina Faso, in 2015 the company Udirba Plus invested in industrial technology performing 10 quality improving functions, including an optical sorter (Tapsoba, 2016). Furthermore, a semi-industrial miller managed by the WendKonta mill had a capacity of 16,500 tons of paddy per year (VECO, 2014). In Liberia, the domestic company Fabrar benefited from subsidies from USAID and shareholding from the International Finance Corporation to upgrade its milling technologies (Oxfam, 2018). In Sierra Leone, the domestic private company *Mountain Lion Rice* managed a mill performing the functions of drying, de-stoning, husking and polishing (FFI and GAIN, 2017).

The public sector also financed the building of industrial and semi-industrial mills in Group 2 countries. In Côte d’Ivoire, the NRDS consisted in building 30 industrial mills and renting them to private companies. These mills have a theoretical capacity of processing five tons of paddy per hour, and components performing pre-cleaning, drying, cleaning, de-stoning, weighing, hulling, paddy separating, whitening (for three units), grading and bagging (Soullier et al., 2019). However, only two of these mills were operating in 2019 (Soullier et al., 2019). They were rented to national companies in which LDC and Gain Logis were stakeholders. The Government of Sierra Leone had also purchased two of these mills, but they were not operational (FFI and GAIN, 2017). In Niger, the public company *Riz du Niger* invested in two semi-industrial mills in Tillabéry and Kollo (Fall 2016; RiceHub, 2016; VECO, 2014). Liberia also features a public semi-industrial mill (FFI and GAIN, 2017).

Whereas contract farming is often used by millers in Group 2 to source paddy, vertical integration is rarer. In Ghana, contracts were documented in the Northern, Volta and Upper East regions, which supply 80% of national rice production. Bidzakin et al. (2018) surveyed 350 farmers in these regions (out of an estimated population of about 10,000), and found that 40% of them participated in contracts. In Burkina Faso, in 2017 the industrial mill Urdiba Plus carried out contracts with 140 producers (Sirdey et al., 2018). In Côte d’Ivoire, the two companies that rented industrial mills from the government in 2018 participated in experimental projects supporting contract farming (Soullier et al., 2019). These contracts included support to agricultural practices to improve yields, such as in-row transplanting. In Mali, the mills had also started implementing contracts with farmer cooperatives (Coulibaly and Soullier, 2020). Furthermore, only few mills in countries in Group 2 integrated rice production. The areas reported were 3200 ha in Mali (Coulibaly and Soullier, 2020), 750 ha in Ghana and 1300 ha in Sierra Leone (FFI and GAIN, 2017). In Burkina Faso, the government supported agribusiness investments in agricultural production (Tyrou et al., 2019), but we did not find any evidence of vertical integration by rice millers. Finally, in Benin and Togo, the ESOP business model (*Entreprises de Services et Organizations de Producteurs*) was developed by two NGOs (with acronyms CIDR and ETD) to foster family farmers' inclusion in processing and marketing. The ESOP managed mini rice mills and purchased their products through contracts. Producers had the opportunity to gradually become shareholders in the processing organization (Maertens and Vande Velde, 2017). The contract specified the supply of inputs, and the quantity, price, delivery time and quality of the paddy (Adabe et al., 2019; Maertens and Vande Velde, 2017). In 2015, there were 15 ESOPs in Togo (FFI and GAIN, 2017) and 17 in Benin (ETD, 2016). They purchased paddy from a few hundreds of producers and marketed around 4000 tons of rice.

Group 3 finally includes countries for which we did not find any evidence of investment in rice VC upgrading in the 2009–2019 period, i.e. Guinea, Mauritania, The Gambia and Guinea-Bissau (Table 2). The largest processing units observed in these countries are mini rice mills, which process between one and two tons of paddy per hour (FFI and GAIN, 2017). The 2008 rice import bills in these countries were below 200 million US$, and paddy production was limited, except for Guinea. Most of the countries in this group are endowed with a comparative advantage of demand, which substantially reduces the pressure to upgrade rice VCs.

## 5 Challenges in rice value chain upgrading in West Africa

### 5.1 Vertical coordination

The evidence suggests that vertical coordination is fraught with several challenges, as contract farming in West Africa reached at most 9% of the national population of rice growers in a country (Table 2). First, several upgraded mills were found to be unprofitable because of limited availability of paddy. This was most notably observed in Niger (VECO, 2014), Côte d’Ivoire (Soullier et al., 2019), Senegal (Soullier and Moustier, 2019), and Burkina Faso (Sirdey et al., 2018), and it was reported to constrain industrial millers in other countries as well (FFI and GAIN, 2017). Contract farming and vertical integration are then deployed to source reliable volumes and quality of paddy. This contrasts with more productive rice export VCs in Asia, where contract farming is predominantly used for governing quality (Ba et al., 2019). Contract farming in domestic VCs in West Africa faces similar challenges as in export VCs in Asia, though. First, contract terms must be tailored to farmers' preferences. This particularly includes the timing of input delivery and payment (Arouna et al., 2017b; Ochieng et al., 2017; Van den Broeck et al., 2017). Second, contracts must improve farmers' living conditions to secure their participation (Barrett et al., 2012). In the case of export VCs of high value products, the literature documents that most contract schemes improve farmers income (Reardon et al., 2009). Some of these positive impacts also extend to the case of rice export VCs (Nhan, 2019) and even to domestic rice VCs in West Africa, e.g., in Ghana (Bidzakin et al., 2018), Côte d’Ivoire (Chiapo, 2017), and Nigeria (Awotide et al., 2015). However, recent evidence in Senegal uncovered a case where contract farming generated zero or negative impact because some millers in an oligopsony position offered contracts to indebted producers that specified lower prices than the market price (Soullier and Moustier, 2018). Impact on livelihoods is closely related to farmers' bargaining power and inclusiveness of smallholders. Recent evidence in rice export VCs suggests that contract farming's typical scale bias towards larger farms can be successfully reduced through policies that encourage horizontal coordination between farmers (Ba et al., 2019). Third, contracts should secure paddy quality. They often specify quality criteria, but the paddy supplied does not always comply with these standards. This was reported in the case of Nigeria (Awotide et al., 2015) and Côte d’Ivoire (Soullier et al., 2019). Fourth, contract farming does not always successfully reduce opportunistic behavior by farmers and millers. For instance, farmers sometimes default on reimbursing the credit when they find another purchaser, and millers can pay farmers a lower price than the one decided at contract signature (Awotide et al., 2015). Identifying adapted enforcement institutions is found to be a critical issue for the reduction of opportunism in rice export VCs (Ba et al., 2019). The supply chain literature documents that farmer price incentives are a major driver to reduce side selling (Barrett et al., 2012). Because contract farming can fail, millers can decide to directly control rice growing (vertical integration). However, they can have restricted access to land, such as in Côte d’Ivoire (Soullier et al., 2019) or their land acquisition can generate conflicts with family farmers (Soullier et al., 2018). This is consistent with the limited evidence on adoption of vertical integration, which covered at most 2% of the national rice area in a country (Table 2).

### 5.2 Managing new technologies

Investments in industrial milling are sometimes undertaken by actors who lack experience in managing the new technologies. The technological change requires millers to develop skills to master equipment and infrastructure, which involves hiring staff and properly training them. Local availability of experts and support services to maintain the equipment and manage the plant are therefore crucial challenges (IFPRI, 2014). Furthermore, unavailability and lack of local markets for spare parts of imported milling equipment can hamper proper technology maintenance and provoke milling breakdowns. This situation is in contrast with global supply chains which are usually close to equipment fabricators. Asian millers are directly supplied in the countries by equipment fabricators such as Alibaba in Thailand. Similarly, larger rice mills prioritize education, training, technology, and innovation (Horadal, 2019). However, global supply chains are still dominated by many small rice mills located in regions where the quality of education and educational opportunities are limited (Horadal, 2019; World Bank, 2013).

### 5.3 Finance

Finance is a major constraint hampering domestic rice VC upgrading. Financing rice growing is a common constraint among family farmers, which can explain their preference for contract farming. However, millers face similar constraints. They rarely have access to formal credit from banks and rely on their own savings, particularly when they are domestic actors. However, significant operational funds are required to invest and collect sufficient paddy to reach profitability. Limited availability of working capital was for instance documented in Niger, where between 2009 and 2013, the RINI company processed volumes that represented between 7% and 31% of its annual capacity (VECO, 2014). It was also reported in Côte d’Ivoire (Soullier et al., 2019), where the AMC group shifted from contract farming to custom milling due to cash-flow constraints. This explains both the low rate of contract offers and, hence, contract participation among rice farmers and the limited adoption of vertical integration mentioned before (Table 2). This is another difference with export VCs, that benefit from support from financial organizations (Ba et al., 2019), and enable importers to offer credits to rice wholesalers. On the contrary, credit arrangement between actors in upgraded domestic VCs is rare. This can explain wholesalers' preference for marketing imported rice. This comparative advantage of imported rice even extends to retail and consumers (Lancon et al., 2004).

### 5.4 Developing consistent policies for a heterogeneous milling sector

Milling technologies are heterogeneous in scale and in their ability to compete against imports in terms of quality and cost. Modern rice VCs governed by industrial and semi-industrial mills supply high quality rice, while traditional rice VCs have more difficulties in supplying quality rice, particularly in terms of homogeneity and purity. The different types of VCs supply different market segments and, hence, contribute differently to food security and income generation. Policy makers face the challenge of creating an enabling environment that encourages equitable and inclusive VC upgrading that benefits large-scale VC entrepreneurs as well as smallholders.

## 6 Conclusion

We compile and review evidence on investment in rice VC upgrading in 15 West African countries over the last decade 2009–2019 in the wake of the world food price crisis. We observed an emerging trend of investment in semi-industrial or industrial milling technologies and the implementation of vertical coordination modes. The evidence revealed that in 2019, there were 57 rice mills with semi-industrial or industrial technologies with an aggregate capacity of 315 tons per hour operating in West Africa. Although contract farming was carried out in eight countries, and millers integrated rice growing in five countries, contract farming reached at best 9% of the national populations of rice growers and vertical integration covered at most 2% of the national rice areas. The latter suggests that these institutional innovations face several challenges and, as a result, are only emerging slowly in response to the competitive pressure experienced by rice VC actors.

Using aggregate upgraded milling capacity as an outcome indicator, we observe substantial heterogeneity in rice VC upgrading among the 15 countries, 89% of which can be explained through three factors. Rice VC upgrading was found to be more dynamic in countries featuring (i) high paddy production, (ii) high import bills and (iii) limited comparative advantage in demand. Rice VC upgrading was found to be dynamic in Nigeria and Senegal, which crowd in the highest investment levels from foreign and domestic private sectors. VC upgrading was found to be moderate and emerging in Ghana, Mali, Côte d’Ivoire, Burkina Faso, Liberia, Niger, Sierra Leone, Benin and Togo. Our review of the literature, consultation with experts and validation with public and private sector actors did not yield any evidence of rice VC upgrading in Guinea, Mauritania, The Gambia and Guinea-Bissau.

Consistent with earlier recommendations (Demont, 2013; Demont et al., 2017; Demont and Ndour, 2015), coastal countries with limited comparative advantage in demand (Nigeria and Senegal) have indeed managed to crowd in the highest investment—including FDI—in rice VC upgrading, while landlocked countries (Mali, Burkina Faso and Niger) and particularly coastal countries with a comparative advantage in demand (Côte d’Ivoire, Sierra Leone, Guinea, The Gambia and Guinea-Bissau) have lagged behind. However, for the latter two groups there is no reason for complacency as their rice sectors are continuously exposed to import competition as well, and will need to maintain quality-based competitiveness before consumers have the time to develop preferences for imported rice (Demont, 2013). In particular, we found that landlockedness does not significantly slow down investment in VC upgrading, exemplified by the emerging evidence in industrial and semi-industrial mills in Mali and Burkina Faso. On the other hand, we found that limited comparative advantage in demand is merely an enabling factor which enables drivers like import bills and paddy supply triggering investment in VC upgrading. Examples are Ghana, Liberia, Benin and Togo, where VC upgrading is emerging despite smaller import bills and paddy production levels compared to Nigeria and Senegal. We acknowledge that our review of the literature, consultation with experts and validation with public and private sector actors may have missed out investments that were not yet reported in the literature or in the authors' professional networks, though.

The first generation of NRDS was heavily focused on productivity and it was argued that more investment in quality upgrading is needed to help domestic rice compete with imported rice in import-biased markets (Demont, 2013). However, our evidence suggests that *productivist* policies aiming at increasing paddy production also indirectly contribute to VC upgrading. Investment in breeding, agronomic and post-harvest yield increase and land extension therefore needs to go hand in hand with investment in VC upgrading to scale up production of quality rice that is able to compete against imported rice and, hence, integrate domestic rice VCs in global markets.

Finally, our assessment can help policy makers and VC actors compare the state of rice VC upgrading among 15 West African countries, learn from more advanced countries that have successfully crowded in private investment and even FDI, and revisit and refine upgrading strategies and policies during the revision of the NRDS under the CARD Phase 2. Our review uncovered several challenges in scaling of rice VC upgrading. We recommend future research to focus on developing optimal policies, coordination and finance mechanisms and efficient technology support chains to help domestic rice VCs compete against imports in terms of quality, efficiency, cost and scale.

## Declaration of competing interest

None.

## Footnotes

1 Other factors that could favour and hinder investment in modern milling technologies that were reviewed include for example World Bank's Doing Business Score (https://www.doingbusiness.org), number of conflicts, Nominal Rate of Protection (NRP) at point of competition (POC), Gross Domestic Product (GDP), average yields (FAO, 2019) and population. However, none of these indicators successfully explained heterogeneity of investment in rice value chain upgrading between the groups. Annual import bills are probably endogenous in that they crowd in investments in VC upgrading that seek to replace imports. Therefore, we chose the 2008 import bill, which is exogenous to the period of our analysis (2009–2019).

2 Côte d’Ivoire can be assumed to feature *genealogical* proximity to the primary center of origin of rice domestication as it hosts the Mandé ethnic group who were the first domesticators of African rice who subsequently migrated southward (Demont et al., 2017).
